# Sarcomatoid hepatocellular carcinoma: From clinical features to cancer genome

**DOI:** 10.1002/cam4.4162

**Published:** 2021-07-31

**Authors:** Cheng Zhang, Shi Feng, Zhenhua Tu, Jingqi Sun, Tao Rui, Xueyou Zhang, Haitao Huang, Qi Ling, Shusen Zheng

**Affiliations:** ^1^ Department of Surgery the First Affiliated Hospital Zhejiang University School of Medicine Hangzhou China; ^2^ Department of Pathology the First Affiliated Hospital Zhejiang University School of Medicine Hangzhou China; ^3^ Key Laboratory of Combined Multi‐Organ Transplantation Ministry of Public Health Hangzhou China

**Keywords:** genome, sarcomatoid hepatocellular carcinoma, survival, therapy

## Abstract

**Background:**

Sarcomatoid hepatocellular carcinoma (HCC) is a rare and highly lethal histological subtype of HCC, with completely unknown genetic etiology and therapeutic targets.

**Methods:**

We included 16 patients with sarcomatoid HCC receiving radical resection among 6731 cases of pathological confirmed HCC in year 2008 to 2018 in our hospital. We compared the clinical features, prognosis and cancer genome between 15 sarcomatoid HCC and propensity score‐matched 75 non‐sarcomatoid HCC patients. The other concurrent case was analyzed using phylogenetic tree to assess the tumor heterogeneity and evolution.

**Results:**

Sarcomatoid HCC group showed larger tumor size, more advanced differentiation grade, lower tumor free survival (*p* = 0.038) and overall survival (*p* = 0.001), and sarcomatoid type was an independent risk factor for patient death. Integrating sarcomatoid subtype into AJCC staging could increase the diagnostic curve in predicting patient survival. The cancer genome spectrum showed sarcomatoid HCC group had significant higher mutation rates in CDKN2A, EPHA5, FANCM and MAP3K1. Mutations in CDKN2A significantly reduced tumor‐free and overall survival in sarcomatoid HCC patients. Moreover, 46.6% sarcomatoid HCC patients had druggable mutations in cell cycle pathway genes, which were targeted by Abemaciclib, et al. We also found sarcomatoid and non‐sarcomatoid lesions might originate from a common progenitor but progress differently.

**Conclusion:**

Our cancer genome analysis showed a specific genomic profile of sarcomatoid HCC, which were characterized by a high mutation rate in cell cycle genes particularly CDKN2A. The results indicate CDK4/6 inhibitors including abemaciclib, ribociclib and palbociclib as potential therapeutic targets and may help for therapeutic decision making.

## Graphical abstract

In summary, there is no specific and effective therapies for sarcomatoid HCC. Our cancer genome analysis showed a specific genomic profile of sarcomatoid HCC, which were characterized by a high mutation rate in cell cycle genes particularly CDKN2A. The results indicate CDK4/6 inhibitors including abemaciclib, ribociclib and palbociclib as potential therapeutic targets and may help for therapeutic decision making.

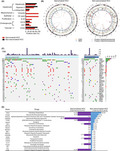

## 1 INTRODUCTION

Hepatocellular carcinoma (HCC) is the sixth most common incident cancer and the fourth leading cause of cancer death worldwide.^1^ Because of high hepatitis B virus prevalence, China has the biggest HCC burden globally (>50%) and HCC is the most common cancer and the leading cause of cancer death in Chinese man under 60 years old.^2^ The treatment strategy has been continuously developed during the last decades including locoregional treatment (e.g., surgery, radiofrequency ablation, and transarterial chemoembolization) and systemic therapy (e.g., tyrosine kinase inhibitors and anti‐PD‐1 antibodies). However, the prognosis remains poor. The median overall survival is around 10 months in advanced HCC treated with systemic therapy.^3^ For those at early stage (single, <5 cm) and received radical surgery, the median survival is still less than 60 months.^4^

HCC is a highly heterogeneous cancer, showing a wide spectrum of pathohistological features and molecular patterns.^5^ Sarcomatoid HCC is a very rare subtype of HCC, with few cases recorded worldwide. The epidemiology, histopathology, radiology, and clinical features of sarcomatoid HCC are largely unknown until the publication of two matched cohort studies in 2019. One included 40 cases of sarcomatoid HCC from the Cancer Registry Database of National Taiwan University Hospital^6^ and another included 102 cases of sarcomatoid HCC from National Cancer Data Base of United States.^7^ Both studies concluded that sarcomatoid HCC was associated with more advanced histological grades compared to non‐sarcomatoid HCC and independently increased the risk of tumor recurrence and mortality. More importantly, there is a lack of effective treatment for sarcomatoid HCC, emphasizing the desideration for novel therapeutic targets.

In this study, we conduct a comprehensive comparison of clinical characteristics and molecular profiles between sarcomatoid HCC and propensity score‐matched non‐sarcomatoid HCC. We perform tumor genome sequencing on tumor samples with matched normal reference DNA to investigate the mutational signatures and explore the druggable targets. We also assess the tumor heterogeneity and tumor evolution over time in a patient with concurrent sarcomatoid HCC and non‐sarcomatoid HCC using phylogenetic analysis.

## 2 MATERIALS AND METHODS

### 2.1 Patients

A total of 6731 cases of HCC were pathological confirmed (histological examination and immunohistochemical staining) between January 2008 and December 2018 in our hospital. Among 6731 cases, 18 were sarcomatoid HCC (0.27%). We excluded 1 case obtained from biopsy and 1 case with combined sarcomatoid HCC and cholangiocarcinoma. Finally, we included 16 cases of sarcomatoid HCC. One patient with concurrent sarcomatoid HCC (segment VII) and non‐sarcomatoid HCC (segment IV) was analyzed separately. The other 15 were compared with propensity score‐matched 75 non‐sarcomatoid HCC (1:5) according to age, gender, and AJCC stage (Figure S1). The study was approved by the Ethics Committee and institutional review board at The First Affiliated Hospital, Zhejiang University School of Medicine, and was in accordance with the Declaration of Helsinki.

### 2.2 Data collection

Patient characteristics including age, gender, primary liver disease, and comorbidities were collected. Serum biochemistries were recorded before surgery. Tumor morphological features including tumor size, number, location, and vascular invasion were recorded according to the imaging (computer tomography and magnetic resonance) and verified by pathology. All patients were routinely followed up in the outpatient clinic and the data were collected.

### 2.3 Immunohistochemistry

All enrolled patients received curative resection. The specimens were formalin‐fixed, paraffin‐embedded, and hematoxylin and eosin (HE)‐stained. Two independent pathologists specialized in gastrointestinal tumor reviewed and confirmed the pathohistological features of all the sections. Sarcomatoid HCC was defined as HCC consisted of sarcomatous portions and sarcomatous elements predominantly composed of spindle‐shaped, pleomorphic, and osteoplastic types of cells.^8^ Immunohistochemical staining was performed using the Envision Detection System (DAKO) according to the manufacturer's instructions. Anti‐CK monoclonal antibody (1:500; LabVision), anti‐CK 7 monoclonal antibody (1:400; LabVision), anti‐CK 19 monoclonal antibody (1:200; LabVision), anti‐hepatocytes monoclonal antibody (1:300; Inyitrogen), anti‐glypican‐3 monoclonal antibody (1:400; Inyitrogen), anti‐α‐fetoprotein monoclonal antibody (1:500; Inyitrogen), anti‐vimentin monoclonal antibody (1:5000; Inyitrogen), and anti‐Ki‐67 monoclonal antibody (1:1000; Inyitrogen) were used.

### 2.4 Tumor genome sequencing

Tumor specimens and matched normal blood were subjected to a 450‐gene panel NGS platform called CSYS assay at a College of American Pathologists (CAP) and Clinical Laboratory Improvement Amendments (CLIA) certified laboratory at OrigiMed. Genomic DNA was extracted using a DNA Extraction Kit (QIAamp DNA FFPE Tissue Kit) according to the manufacturer's protocols. Diluted libraries were sequenced to a mean coverage of 800× for FFPE samples and 300× for matched blood samples on an Illumina Nova‐seq 6000 Platform (Illumina Incorporated). Genomic alterations, including single nucleotide variants (SNVs), short and long insertions/deletions (indels), copy number variations (CNVs), and gene rearrangements, were subjected to advanced analysis. First, reads were aligned to human genome reference sequence (hg19) by Burrows–Wheeler Aligner, and polymerase chain reaction (PCR) duplicates were removed using Picard. Second, SNVs and short indels were identified by MuTect after quality recalibration and realignment using Genome Analysis Toolkit (GATK) and in‐house pipeline. Short indels were then calibrated using the results from Pindel. Synonymous SNVs and known germline polymorphisms in the U.S. National Center for Biotechnology Information's Single Nucleotide Polymorphism Database (dbSNP) were not counted. Read depths were normalized within target regions by Exome Copy number Alterations/Variations annotATOR (EXCATOR). The log‐ratio per region of each gene was calculated, and customized algorithms were used to detect copy number changes. Tumor cellularity was estimated by allele frequencies of sequenced SNPs. A customized algorithm was developed to detect gene rearrangements and long indels.^9^

Reliable somatic alterations were detected in the raw data by comparison with matched blood control samples. At minimum, five reads and minimum variant allele frequency of 1% were required to support alternative calling. For CNVs, focal amplifications were characterized as genes with thresholds >4 copies for amplification and 0 copies for homozygous deletions. For the calling of gene rearrangements, aligned reads with abnormal insert size of over 2000 or 0 bp were collected and used as discordant reads. Next, the discordant reads with a distance less than 500 bp formed clusters that were further assembled to identify potential rearrangement breakpoints. The breakpoints were reconfirmed by the BLAST‐like alignment tool and the resulted chimeric gene candidates were annotated. A subsequent manual review was performed for clinical relevance inference based on literatures and clinical trials.

### 2.5 Phylogenetic analysis

We employed public evolutionary software ‘SciClone’ with default parameters to analyze the clonal structure based on a Bayesian clustering method.^10^ The brief workflow is described as follows: an independent input was used to analyze the clonal structure in one tumor and another tumor for DNA at baseline and matched tissue samples, respective. For serial DNA, multiple SNV inputs of each sample were used to analyze serial clonal population. Cancer cell fraction was calculated with the mean of predicted cellular frequencies. Cluster with the highest mean VAF was identified as the clonal cluster, and mutations in this cluster were clonal mutations.

### 2.6 Statistical analysis

Quantitative and categorical variables were presented as mean ± SD and values (percentages). Student's *t*‐test, Mann–Whitney test, and chi‐squared test were used for variable comparison. Cut‐off values were selected according to both statistical and clinical significances. Kaplan–Meier test with Breslow method was used for survival comparison. Cox proportional hazard method was used for risk factors analysis. Areas under the curve (AUC) were calculated to assess the diagnostic accuracy. SPSS 13.0 (SPSS Inc) was used to complete the analyses. A *p* value of <0.05 was considered as statistical significance.

## 3 RESULTS

### 3.1 Clinical comparison

Patient characteristics are shown in Table 1. Most of the patients were male (86.7%), with hepatitis B virus (HBV)‐related cirrhosis (81.1%) and Child‐Pugh A (96.7%). There was no significant difference between the two groups in age, gender, etiology, and comorbidities. Most of the parameters in lab investigations were comparable between the two groups. But there was a significantly lower serum albumin level in sarcomatoid HCC group than non‐sarcomatoid HCC group (*p* < 0.001).

**TABLE 1:** Patient characteristics

	Sarcomatoid (*n* = 15)	Non‐sarcomatoid (*n* = 75)	*p*
Age (years)	63.9 ± 8.7	64.0 ± 11.7	0.973
Male, *n* (%)	13 (86.7)	10 (86.7)	1.000
Etiology, *n* (%)			
HBV+	10 (66.7)	63 (84.0)	0.117
HBV DNA >10^3^ copy/ml	5 (33.3)	25 (33.3)	1.000
Antivirus before HCC diagnosis	4 (26.7)	27 (36.0)	0.487
Liver function			
Cirrhosis, *n* (%)	10 (66.7)	57 (76.0)	0.449
Child‐Puph A, *n* (%)	14 (93.3)	73 (97.3)	0.431
MELD score	4.8 ± 1.8	5.1 ± 1.0	0.468
Lab investigation			
Albumin (g/dl)	36.9 ± 5.3	43.4 ± 5.6	<0.001
Alanine amiotransferase (U/L)	24.7 ± 8.6	30.8 ± 15.9	0.155
Aspartate aminotransferase (U/L)	27.4 ± 5.7	36.1 ± 20.6	0.109
γ‐Glutamyl Transferase (U/L)	115.6 ± 98.0	96.5 ± 46.0	0.245
Bilirubin (mg/dl)	1.29 ± 1.75	0.89 ± 0.42	0.082
Creatinine (mg/dl)	0.80 ± 0.16	0.86 ± 0.19	0.154
International normalized ratio	1.06 ± 0.06	1.03 ± 0.06	0.056
Platelet count	147.5 ± 76.3	170.9 ± 56.9	0.174
Comorbidities, *n* (%)			
Diabetes	1 (6.7)	8 (10.7)	0.637
Hypertension	2 (13.3)	18 (24.0)	0.364
Dyslipidemia	2 (13.3)	5 (6.7)	0.379

Abbreviations: HBV, hepatitis B virus; MELD, model for end‐stage liver diseases.

The tumor features are listed in Table 2. The ALCC stage was completely matched between the two groups. The BCLC stage, tumor number, tumor location, vascular invasion, α‐fetoprotein level did not differ significantly between the two groups. Sarcomatoid HCC group showed significantly larger tumor size (>5cm, *p* = 0.030) and more advanced differentiation grade (poor and undifferentiated, *p* < 0.001) than non‐sarcomatoid HCC group.

**TABLE 2:** Tumor features

	Sarcomatoid (*n* = 15)	Non‐sarcomatoid (*n* = 75)	*p*
AJCC stage, *n* (%)			1.000
I	6 (40.0)	30 (40.0)	
II	4 (26.7)	20 (26.7)	
III	2 (13.3)	10 (13.3)	
IV	3 (20.0)	15 (20.0)	
BCLC stage, *n* (%)			0.881
0	1 (6.7)	3 (4.0)	
A	8 (53.3)	34 (45.3)	
B	1 (6.7)	6 (8.0)	
C	5 (33.3)	32 (42.7)	
Differentiation grade, *n* (%)			<0.001
Well	0 (0)	2 (2.7)	
Moderate	0 (0)	36 (48.0)	
Poor	8 (53.3)	36 (48.0)	
Undifferentiated	7 (46.7)	1 (1.3)	
Tumor size, *n* (%)			0.086
<2 cm	1 (6.7)	6 (8.0)	
2–5 cm	3 (20.0)	37 (49.3)	
>5 cm	11 (73.3)	32 (42.7)	
Tumor number, *n* (%)			0.120
Single	12 (80.0)	44 (58.7)	
Multiple	3 (20.0)	31 (41.3)	
α‐fetoprotein, *n* (%)			0.200
<20 ng/ml	10 (66.7)	36 (48.0)	
20–400 ng/ml	4 (26.7)	18 (24.0)	
>400 ng/ml	1 (6.7)	21 (28.0)	
Tumor location, *n* (%)			0.673
Left lobe	2 (13.3)	17 (23.0)	
Right lobe	12 (80.0)	51 (68.9)	
Both lobes	1 (6.7)	6 (8.1)	
Vascular invasion, *n* (%)			
Macro‐	3 (20.0)	14 (18.7)	0.904
Micro‐	3 (20.0)	25 (33.3)	0.309
Adjuvant therapy, *n* (%)			
TACE	7 (46.7)	43 (57.3)	0.448
Sorafenib	1 (6.7)	3 (4.0)	0.647

Abbreviation: TACE, Transcatheter arterial chemoembolization.

**FIGURE 1:**
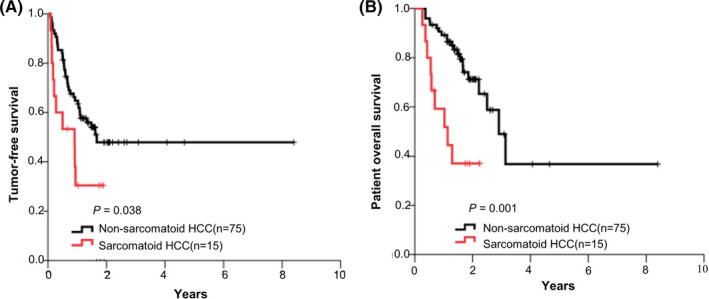
The comparison of survival curves between patients with sarcomatoid HCC and non‐sarcomatoid HCC. (A) tumor‐free survival curves; (B) patient overall survival curves

All patients received radical surgical resection and around 55% had adjuvant therapy with transarterial chemoembolization (TACE). During a follow‐up time of 0.3–8.4 years, sarcomatoid HCC group had significantly lower tumor‐free survival (*p* = 0.038) and overall patient survival (*p* = 0.001) than non‐sarcomatoid HCC group (Figure 1). In patients with sarcomatoid HCC, the 6‐month and 1‐year recurrence rates were 46.7% and 69.5%, the 1‐year and 2‐year cumulative survival rates were 59.3% and 37.0%. In the multivariate Cox hazard model, sarcomatoid type was an independent risk factor for patient death as well as macrovascular invasion (Table 3). In addition, AJCC staging was a valid tool for predicting both tumor recurrence (RR = 1.626, *p* = 0.001) and patient death (RR = 1.825, *p* < 0.001). Integrating sarcomatoid subtype into AJCC staging could sharply increase the AUC from 0.665 (0.539, 0.791) to 0.741 (0.623, 0.858) in predicting patient survival.

**TABLE 3:** Risk factors of patient death

	Univariate	*p*	Multivariate	*p*
	RR (95%CI)		RR (95%CI)	
Sarcomatoid type	3.523 (1.575, 7.877)	0.002	3.853 (1.701, 8.726)	0.001
Tumor size >5 cm	3.602 (1.628, 7.969)	0.002		
Macrovascular invasion	3.470 (1.592, 7.562)	0.002	3.778 (1.710, 8.346)	0.001
Microvascular invasion*	2.269 (1.052, 4.892)	0.037		

* Those with macrovascular invasion was excluded in multivariate analysis.

### 3.2 Molecular comparison

**FIGURE 2:**
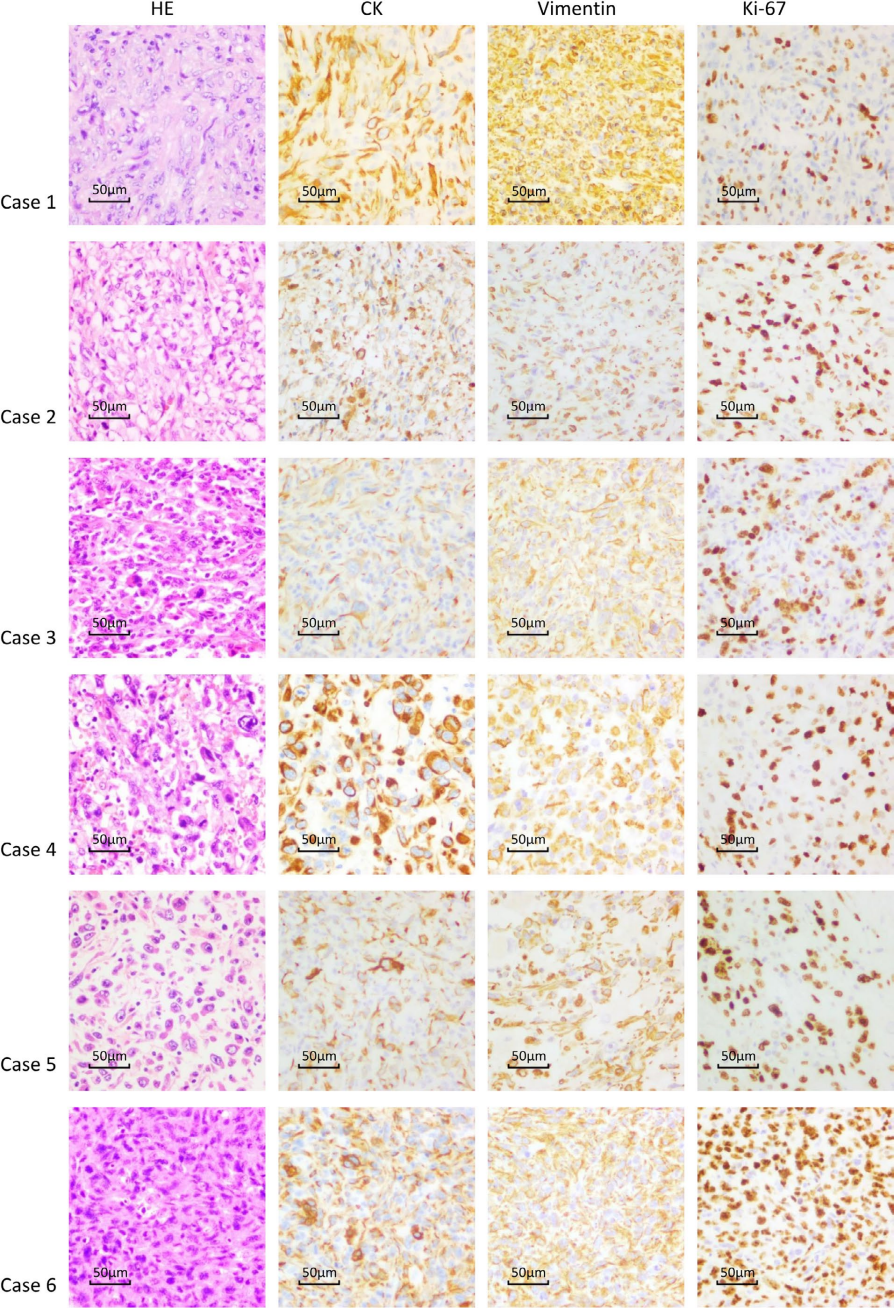
The histopathological presentation of sarcomatoid HCC (case 1–6)

**FIGURE 3:**
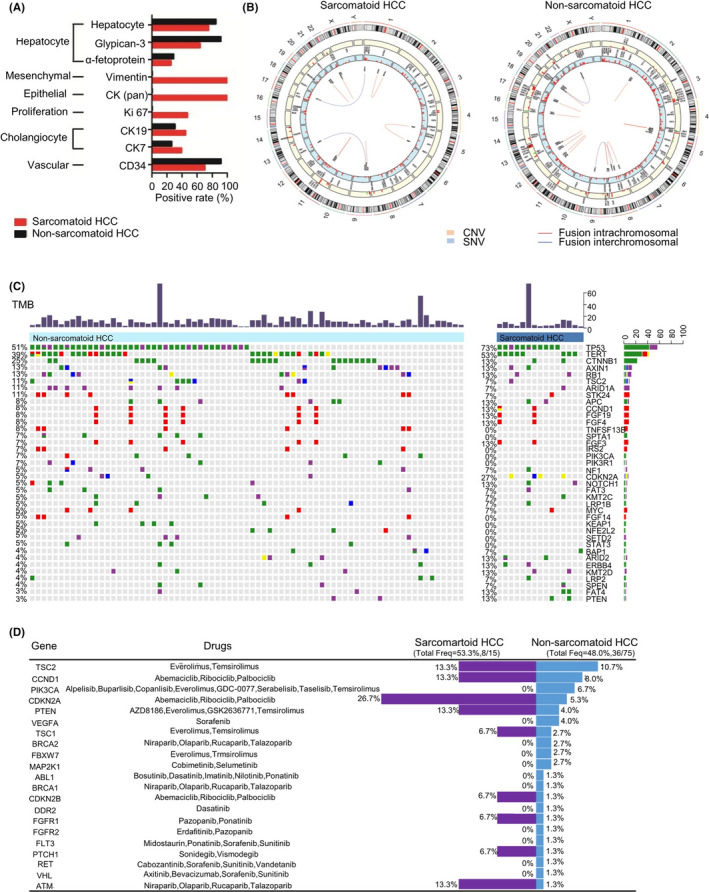
The molecular diversity between sarcomatoid HCC and non‐sarcomatoid HCC. (A) The comparison of immunohistochemistry markers; (B) The overview of clinically relevant genomic alterations in two groups; (C) The differential genomic alterations between the two groups; (D) The comparison of druggable mutations

The histopathological feature of all 15 cases of sarcomatoid HCC is presented in Figure 2 and Figure S2. In immunohistochemistry test, compared to non‐sarcomatoid HCC, sarcomatoid HCC showed comparable rates of positive conventional HCC markers (hepatocytes, glypican‐3, and α‐fetoprotein), higher rates of positive cholangiocyte markers (CK7 and CK19), and specific expression of mesenchymal/epithelial markers (vimentin and CK) (Figure 3A). The median value of cellular proliferation marker ki67 was 50% (range: 25–80%) in six sarcomatoid HCC samples.

The cancer genome spectrum was further evaluated by NGS. A total of 386 and 960 genetic variants including SNVs, CNVs, and INDELs, were detected in 15 cases of sarcomatoid HCC and 75 cases of non‐sarcomatoid HCC, respectively, in which 169 and 537 were clinically relevant genomic alterations (Figure 3B). The most commonly mutated genes were *TP53* (50.7%), *TERT* (38.7%), and *CTNNB1* (25.3%) in non‐sarcomatoid HCC and *TP53* (73.3%), *TERT* (53.3%), and *CDKN2A* (26.6%) in sarcomatoid HCC (Figure 3C). Sarcomatoid HCC showed significant higher mutation rates in *CDKN2A* (26.6% vs. 4.0%, *p* = 0.024), *EPHA5* (13.3% vs. 0%, *p* = 0.026), *FANCM* (13.3% vs. 0%, *p* = 0.026), and *MAP3K1* (13.3% vs. 0%, *p* = 0.026) than non‐sarcomatoid HCC. Mutation in *EPHA5*, *FANCM*, and *MAP3K1* were not identified in our non‐sarcomatoid HCC cohort. The mutational signature showed a higher mutation frequency in cell cycle pathway in sarcomatoid HCC group than that in non‐sarcomatoid HCC group (86.7% vs. 61.2%, *p* = 0.077). In addition, genomic alterations of *CDKN2A* identified in sarcomatoid HCC were rearrangement (3/4) and gene homozygous deletion (1/4), which would lead to the loss of function, while substitutions or short indels were the variation types identified in non‐sarcomatoid HCC cohort. The four sarcomatoid HCC patients with *CDKN2A* mutation suffered HCC recurrence with a median time of 2.4 months after surgery and had significantly lower survival as compared to the other 11 sarcomatoid HCC patients without *CDKN2A* mutation (Figure S3).

We also compared the common genetic variants between the sarcomatoid HCC group and some large HCC cohorts from The Cancer Genome Alters research network (TCGA)^11^ and Memorial Sloan Kettering Cancer Center (MSKCC)^12^ (Table 4). The above findings that sarcomatoid HCC showed significantly higher mutation rates in *CDKN2A*, *EPHA5*, *FANCM*, and *MAP3K1* were further validated in both cohorts.

**TABLE 4:** Comparison of genetic variants between sarcomatoid HCC and non‐sarcomatoid HCC

(%)	Sarcomatoid HCC (*n* = 15)	Non‐sarcomatoid HCC (*n* = 75)	TCGA (HCC) (*n* = 363)	MSKCC (HCC) (*n* = 105)
*TP53*	73	51	22	24
*TERT*	53	39	44*	40
*CTNNB1*	13	25	36	30
*CDKN2A*	27	5	3	1
*EPHA5*	13	0	1	3
*FANCM*	13	0	2	0
*MAP3K1*	13	0	1	2

* TERT promoter mutations were investigated in 196 patients.

To identify potential targets for precision and effective therapy, we further analyzed the druggable mutations. There were 8 (53.3%) patients with potential druggable targets in sarcomatoid HCC group and 36 (48.0%) in non‐sarcomatoid HCC group (*p* = 0.924, Figure 3D). Among 15 sarcomatoid HCC patients, 7 (46.6%) had genetic variants in cell cycle pathway genes (4 *CDKN2A*, 1 *CDKN2B*, and 2 *CCND1*), which was significantly higher than that (14.7%, 11/75) in non‐sarcomatoid HCC patients (*p* = 0.013). The genetic variants were targeted by abemaciclib, ribociclib, and palbociclib.

CNVs were detected in 42/75 (56.0%) patients with non‐sarcomatoid HCC and in 7/15 (46.7%) patients with sarcomatoid HCC (*p* = 0.705). Gene loss in chromosome 9 (1/15, 6.7%) and 22 (1/15, 6.7%) and gain in chromosome 4, 8, 9, 11, 13, 14, 20, and 22 (6/15, 40.0%) were observed in sarcomatoid HCC. Genes loss in chromosome 2, 3, 4, 5, 9, 10, 13, 15, and 17 (16/75, 21.3%) and gain in chromosome 1, 2, 3, 5, 6, 8, 9, 10, 11, 13, 14, 15, 16, 17, 19, 20, 22, and X (38/75, 50.7%) were identified in non‐sarcomatoid HCC (Figure S4).

*TP53*‐*TERT* aberrations were the most commonly detected co‐mutations (16/75, 21.3%) in non‐sarcomatoid HCC group, while (5/15, 33.3%) patients harbored *TP53*‐*TERT* co‐mutations in sarcomatoid HCC group (*p* = 0.316). We detected 29 and 27 paired concomitant aberrations with statistical significance in sarcomatoid HCC group and non‐sarcomatoid HCC group (Figure S5).

In addition, the median values of tumor mutation burden were 6.1 muts/Mb (range: 0.7–48.4) and 6.9 muts/Mb (range: 2.5–75.9) in sarcomatoid HCC and non‐sarcomatoid HCC groups. There was no significant difference between the two groups. Microsatellite status was evaluated in 69 patients and no instability‐high was identified.

**FIGURE 4:**
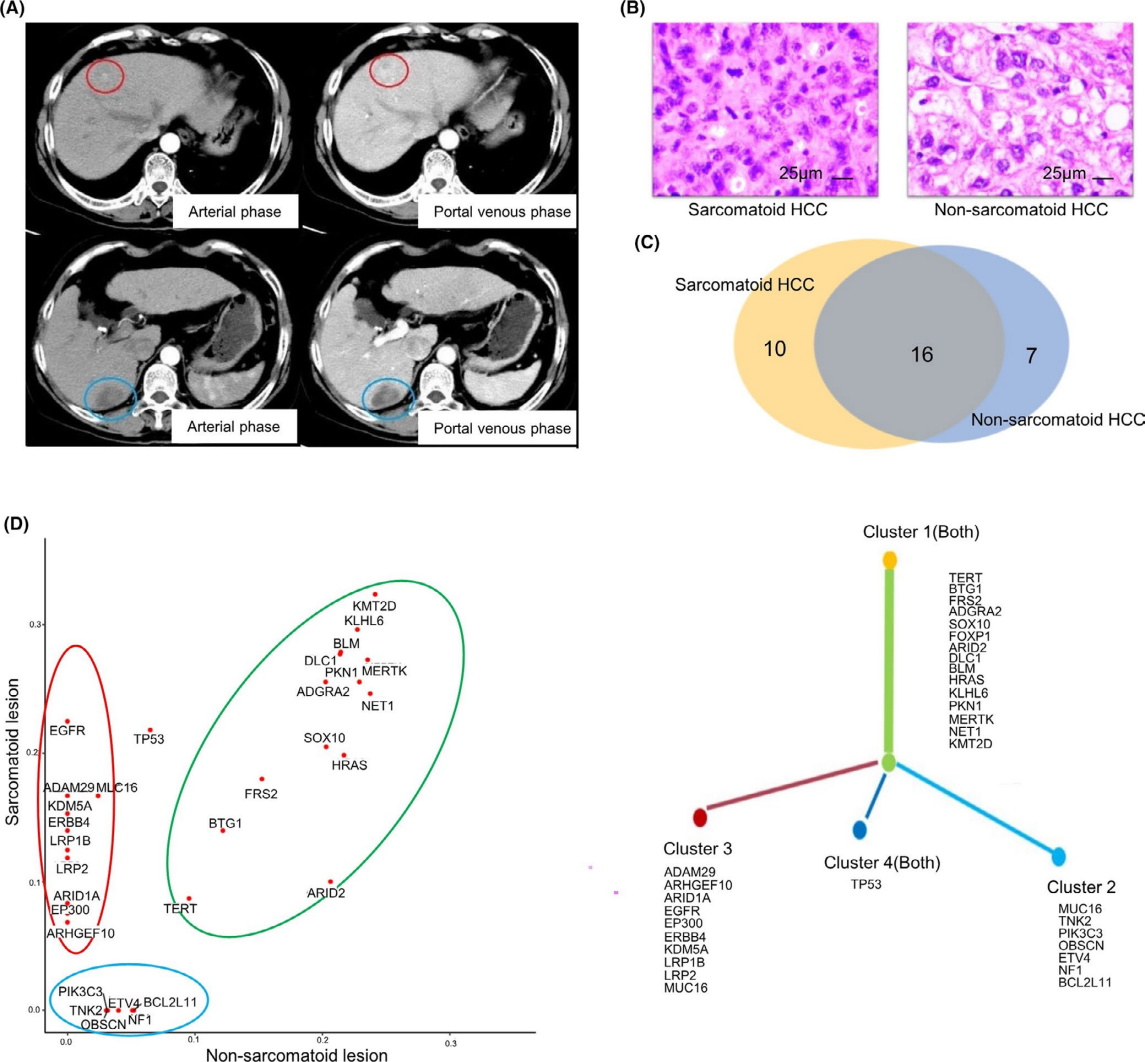
The phylogenetic analysis in a patient with concurrent sarcomatoid HCC and non‐sarcomatoid HCC. (A) The computer tomography scan of sarcomatoid HCC (segment VII, blue circle) and non‐sarcomatoid HCC (segment IV, red circle); (B) The hematoxylin and eosin (HE)‐staining; (C) The overview of somatic variants in the two lesions; (D) The phylogenetic tree

We further investigated the tumor heterogeneity and tumor evolution over time in a patient with concurrent sarcomatoid HCC (segment VII) and non‐sarcomatoid HCC (segment IV) (Figure 4A and 4B). There were 7 (30.4%) and 10 (38.5%) unique somatic variants (SNVs and INDELs) in non‐sarcomatoid lesion and sarcomatoid lesion, respectively (Figure 4C). There were also 16 overlapping variants. Then a phylogenetic tree was created with somatic mutations (Figure 4D). Sarcomatoid HCC was segregated into three clusters (clusters 1, 3, and 4), and non‐sarcomatoid HCC formed cluster 1, 2, and 4. Based on the evolutionary tree, the two tumors shared common mutations in clusters 1 and 4, suggesting a common tumor initiation. The two tumors also presented specific mutations, clusters 2 and 3. Clusters 3 diverged earlier than cluster 2, indicating different tumor differentiation and progression.

## 4 DISCUSSION

This study confirmed that sarcomatoid HCC is a very rare and highly malignant subtype of HCC. In 6731 patients with pathological proved HCC during 2008–2018, 18 were diagnosed as sarcomatoid HCC. The cumulative incidence of sarcomatoid HCC was 0.27%, which was consistent with two previous studies (0.09–0.79%).^6^, ^7^ Because of the rarity, we performed a matched cohort analysis to reveal the clinical characteristics and genetic features of sarcomatoid HCC. We firstly performed prognosis analysis in the study cohort and found out old age and advanced AJCC stage were independent risk factors. In addition, 80% were male patients, to avoid potential gender bias, we employed it in the propensity match. Compared with age, gender, and AJCC stage strictly matched non‐sarcomatoid HCC, sarcomatoid HCC showed significantly larger tumor size and poorer pathological differentiation grade. The result was further proved by another two matched non‐sarcomatoid HCC cohorts. All enrolled patients received radical surgical resection in this study. Patients with sarcomatoid HCC had significantly lower tumor‐free survival and overall patient survival than those with non‐sarcomatoid HCC group during a follow‐up time of 0.3–8.4 years. About half of patients with sarcomatoid HCC suffered tumor recurrence within 6 months and died during the first year after radical surgical resection. Of note, sarcomatoid subtype independently increased the risk of tumor recurrence and patient death after surgery. The predictive efficacy of AJCC staging system on patient prognosis could be greatly improved by integrating it with sarcomatoid subtype. Therefore, sarcomatoid variant is a highly aggressive subtype of HCC and needs to be paid more attention.

There is still a lack of effective therapeutic strategy for sarcomatoid HCC. As we described above, the prognosis was dismal even though patients received radical surgical resection. Whether liver transplantation could achieve a better outcome than resection remains unclear. Ling et al.^13^ performed living donor liver transplantation for three patients with early stage sarcomatoid HCC (single tumor <3 cm, normalα‐fetoprotein level, and without microvascular invasion). Out of the three patients, two developed tumor recurrence within 6 months after transplantation, the other one (tumor size 1.0 cm) did not suffer recurrence during a 12.3 months follow‐up. Hwang et al.^14^ performed living donor liver transplantation for four cases of sarcomatoid HCC, 3 within and 1 without Milan criteria. The 1‐year recurrence rate and survival rate were 50% and 75%, respectively. Both studies concluded that sarcomatoid HCC is unfavorable tumor histology for liver transplantation. In addition, tyrosine kinase inhibitors such as sorafenib^15^ and sunitinib^16^ seem to have failed in treating sarcomatoid HCC. Therefore, novel and effective therapeutic strategies need to be developed.

The molecular pathogenesis of sarcomatoid HCC remains unknown. Kodama T et al. identified cancer‐related genes such as *MET*, *GAB1*, and *HECT* that may drive epithelial‐mesenchymal transition in HCC.^17^ Morisue R et al. detected the transcriptome in two sarcomatoid HCCs and five non‐sarcomatoid HCC and found upregulation of genes that associated with epithelial‐to‐mesenchymal transition and inflammatory responses in sarcomatoid HCC.^18^ In our study, we found distinct genetic patterns between sarcomatoid HCC and non‐sarcomatoid HCC. The major finding was that sarcomatoid HCC had a high rate of rearrangement and homozygous deletion in *CDKN2A* gene, which leads to a loss of gene function. *CDKN2A* is a well‐known tumor suppressor gene, which encodes the p16 protein and plays a pivotal role in cell cycle through the regulation of the cyclin‐dependent kinase (CDK) 4/6 and cyclin D complexes.^19^ Genetic and epigenetic aberrations of *CDKN2A* lead to enhanced carcinogenesis and poor prognosis in various cancer types including lymphoma, skin cancer, ovarian cancer, and prostate cancer.^19^ In HCC, the abnormalities in *CDKN2A* gene and cell cycle pathway could promote cell proliferation and tumor growth.^20^, ^21^, ^22^ Therefore, we assumed that sarcomatoid HCC had a larger tumor size than non‐sarcomatoid HCC, which was reported in this study and the previous ones,^6^, ^7^ maybe at least partially due to the *CDKN2A* mutation.

More importantly, the inactivation of cell cycle pathway through *CDKN2A*/*B* loss could be a potential druggable target for sarcomatoid HCC. It has been reported that *CDKN2A*/*B* loss is significantly associated with the response to CDK4/6 inhibitors including palbociclib, ribociclib, and abemaciclib.^23^, ^24^ A recent study showed that ribociclib had a potent anti‐proliferation effect via cell cycle arrest in HCC cell lines with low p16 protein content.^25^ Moreover, ribociclib showed a synergistic interaction with sorafenib in HCC cells.^25^ In this study, nearly half of sarcomatoid HCC (7/15) were detected druggable mutations that are potentially sensitive to CDK4/6 inhibitors. In addition, more evidences for target therapy could be accumulated from sarcomatoid variant of other cancer types. For instance, *CDKN2A* is the TOP3 mutated genes in sarcomatoid renal cell carcinoma (7/26, 26.9%)^26^ and also has a relative high mutation rate in sarcomatoid carcinoma of lung (4/24, 16.7%).^27^ FISH detection of *CDKN2*A (p16) homozygous deletion is an effective way to evaluate sarcomatoid component in mesothelioma.^28^ Therefore, CDK4/6 inhibitors may be considered a potential treatment option for sarcomatoid HCC, either alone or in combination with the first line target therapy such as sorafenib and lenvatinib.

Besides *CDKN2A*, another three genes including *EPHA5*, *FANCM*, and *MAP3K1* showed significantly higher mutation rates in sarcomatoid HCC than non‐sarcomatoid HCC. Both *EPHA5* and *FANCM* are considered as tumor suppressors. *EPHA5* encoding protein belongs to the largest receptor tyrosine kinases subfamily EPH. EPHA receptor kinases participate in various cellular processes such as cell morphology maintenance, adhesion, migration, proliferation, and differentiation.^29^ Loss of EPHA5 expression was associated with low tumor histological grade and poor patient outcome in various cancer types.^30^, ^31^ *FANCM* encodes a conserved and structure‐specific DNA translocase. Loss‐of‐function mutations within the *FANCM* Gene increase the susceptibility of breast and ovarian cancer.^32^ In addition, mutations in *MAP3K1* are associated with sensitivity to MEK inhibitors in multiple patient‐derived xenograft (PDX) tumor models.^33^ However, the above results were largely limited by the sample size.

In this cohort, there was a patient with concurrent sarcomatoid and non‐sarcomatoid lesions in the liver. We performed a phylogenetic comparison of sarcomatoid lesion to non‐sarcomatoid lesion in this case. Most of the somatic variants were overlapped between the two lesions. In addition, the two lesions shared common mutations in the trunk of phylogenetic tree, indicating that they had the same origin. However, the branch of phylogenetic tree showed the different progression of the two tumors and suggest that sarcomatoid lesion may appear more preferentially, which provides insights for why sarcomatoid HCC has a worse prognosis than non‐sarcomatoid HCC. We are collecting more cases with concurrent sarcomatoid and non‐sarcomatoid lesions to support the results by statistical analysis.

There were limitations. First, the sample size was small, resulting in limited statistical power particularly in the genetic variants analysis. Second, racial difference such as the high mutation rate in *TP53* gene in Asian HCC cohort^34^ was beyond the scope of this study and needed to be further explored. Third, the potential therapeutic value of CDK4/6 inhibitors was theoretically identified in this study. But the effect needs to be verified in clinical cases.

In summary, sarcomatoid variant is an unfavorable form of HCC with dismal prognosis. At present, there is no specific and effective therapies for sarcomatoid HCC. Our cancer genome analysis showed a specific genomic profile of sarcomatoid HCC, which were characterized by a high mutation rate in cell cycle genes particularly *CDKN2A*. The results indicate CDK4/6 inhibitors including abemaciclib, ribociclib, and palbociclib, as potential therapeutic targets and may help for therapeutic decision making.

## CONFLICT OF INTEREST

The authors declare no conflicts of interest that pertain to this work.

## ETHICAL APPROVAL STATEMENT

This study was approved by the Ethical Committee of the First Affiliated Hospital, Zhejiang University School of Medicine.

## INFORMED CONSENT STATEMENT

The patients provided their written informed consent to participate in this study.

## Supporting information

Fig S1‐S5

## Data Availability

The data that support the findings of this study are available on request from the corresponding author. The data are not publicly available due to privacy or ethical restrictions.
