# Supplementary material for: Sarcomatoid hepatocellular carcinoma: From clinical features to cancer genome
Source: Cancer Med. 2021 Jul 31;10(18):6227–38. doi: 10.1002/cam4.4162 (PMC8446410; doi:10.1002/cam4.4162)
Supplement: Supplementary file 1 — Fig S1‐S5 [file CAM4-10-6227-s001.pdf]

Supplemental figures

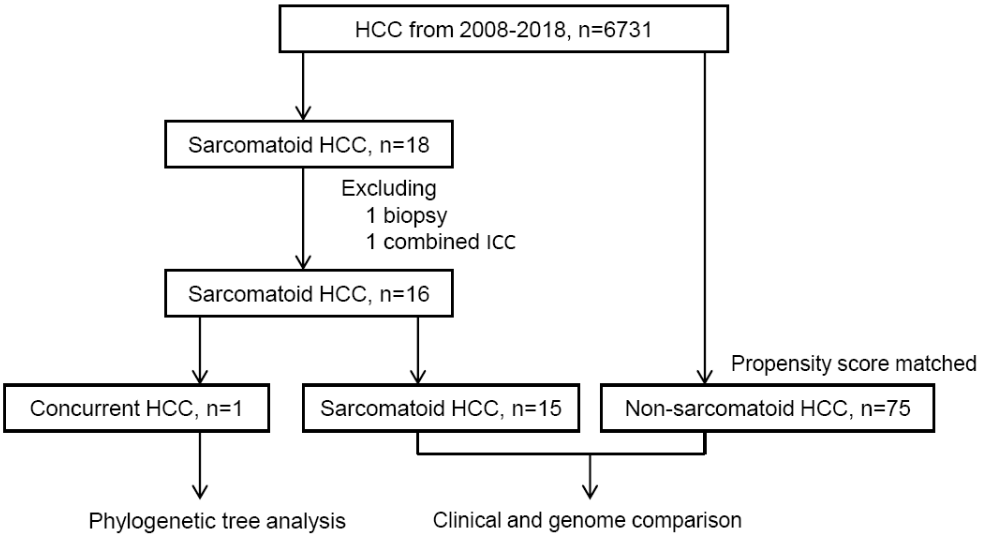

Fig. S1. Flow chart of patient selections.

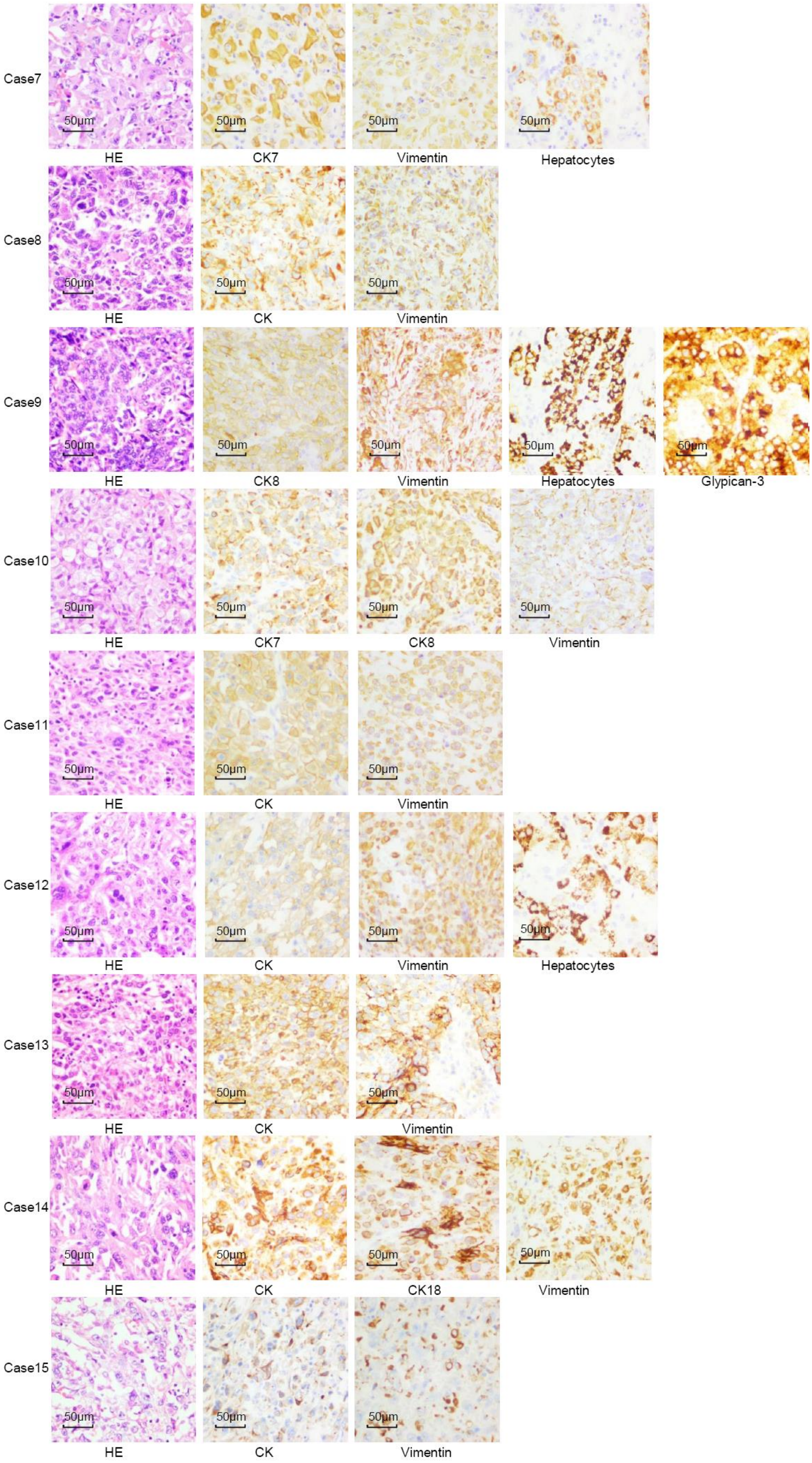

Fig. S2. The histopathological presentation of sarcomatoid HCC (case 7-15).

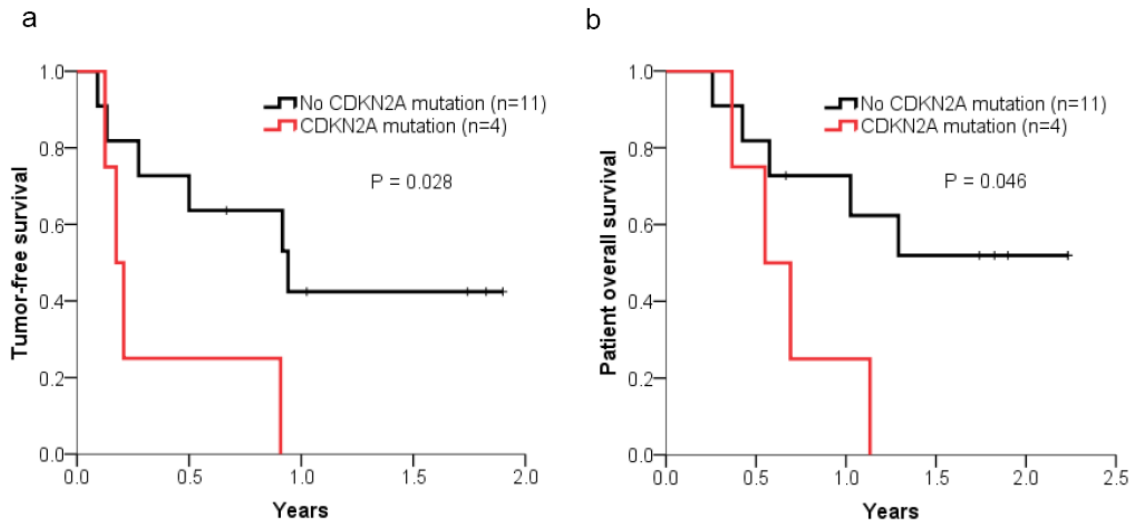

Fig. S3. The comparison of survival curves between sarcomatoid HCC patients with and without *CDKN2A* mutation. **a** tumor-free survival curves; **b** patient overall survival curves.

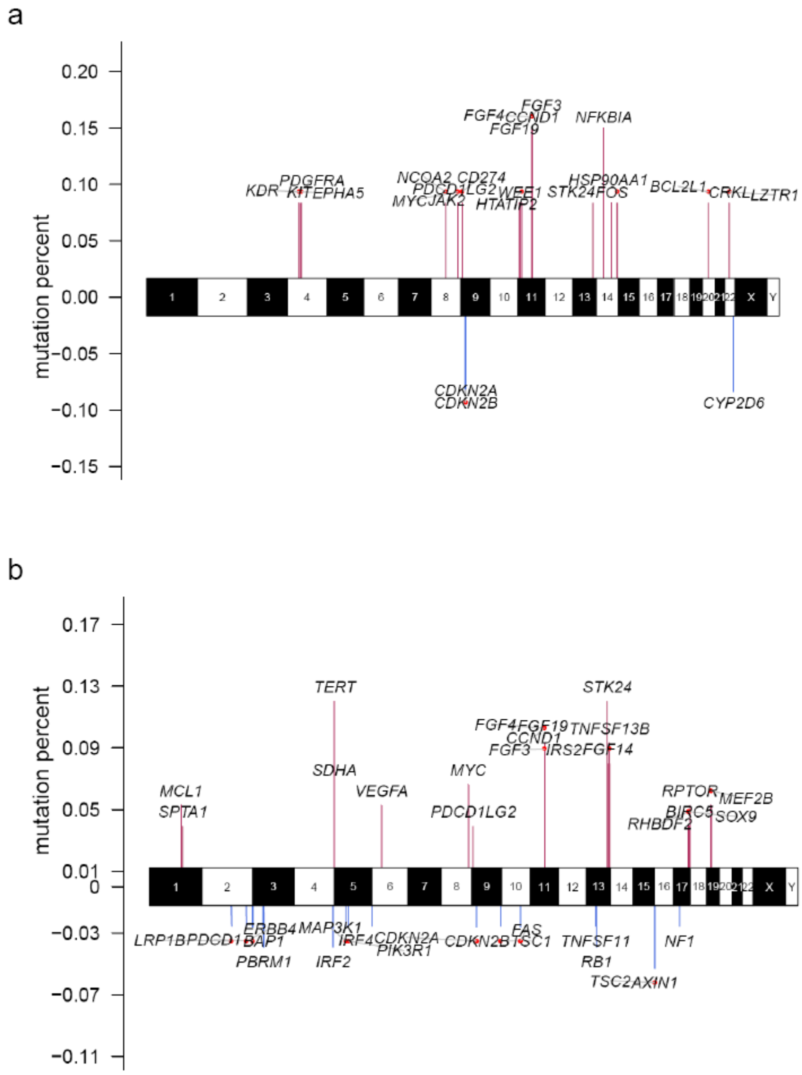

Fig. S4. Copy number variants in sarcomatoid HCC (**a**) and non-sarcomatoid HCC (**b**).

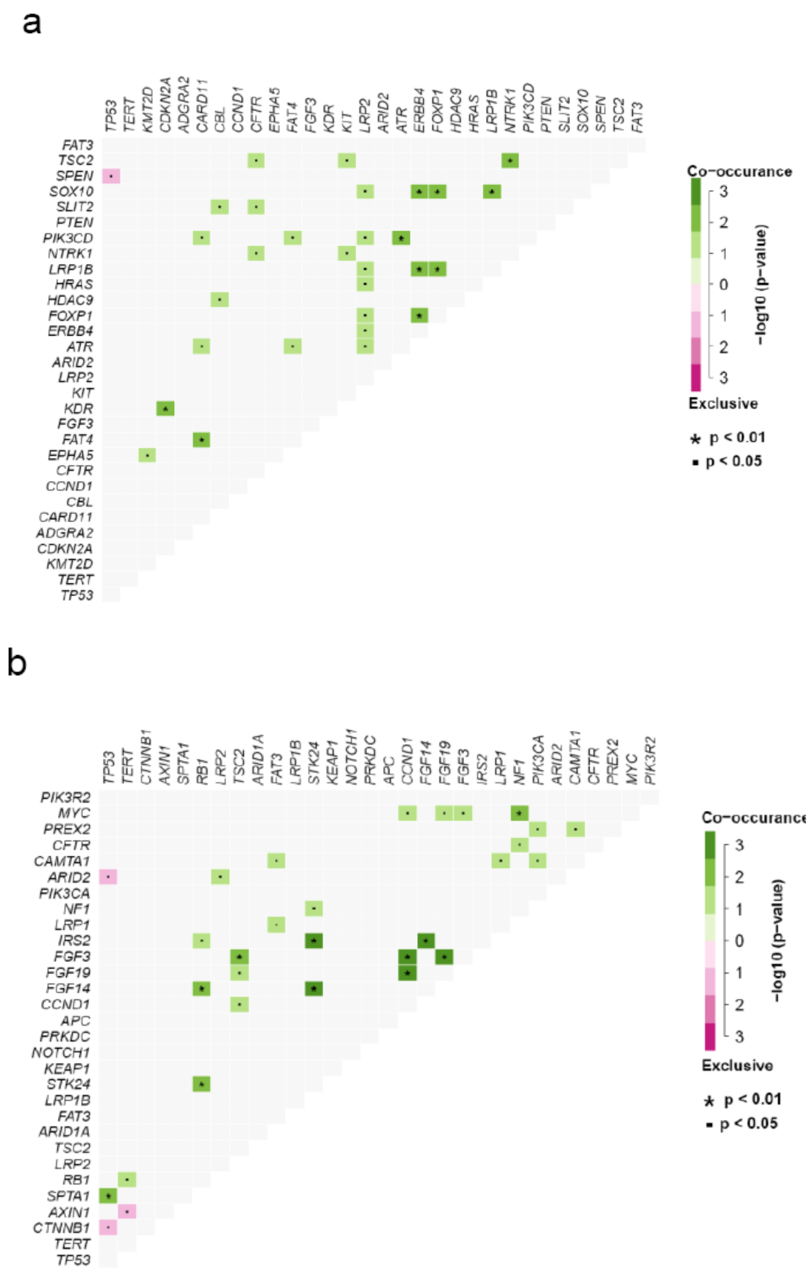

Fig. S5. Concomitant genomic alterations detected in sarcomatoid HCC (**a**) and non-sarcomatoid HCC (**b**).
